# Differential Associations of Authoritarian Submission and Traditionalism with Fake-News Detection and Conspiracy-Claim Endorsement: Evidence from Two Romanian Samples in the 2024–2025 Electoral Cycle

**DOI:** 10.3390/bs16091490

**Published:** 2026-08-26

**Authors:** Teodor Răileanu-Olariu, Adriana Mutu

**Affiliations:** 1 Faculty of Psychology and Educational Sciences, Alexandru Ioan Cuza University of Iași, 700554 Iași, Romania; 2 Department of Humanities & Market Research, ESIC Business & Marketing School, 08021 Barcelona, Spain; adriana.mutu@esic.edu

**Keywords:** right-wing authoritarianism, conspiracy theories, fake news, submission, traditionalism, conspiracy-claim endorsement, misinformation, media literacy, presidential elections, Romania

## Abstract

Susceptibility to misinformation-related content threatens democratic processes, yet the roles of individual authoritarianism components remain understudied. We report two independent Romanian studies conducted during the 2024–2025 electoral cycle, examining associations of aggression, conventionalism/traditionalism, and submission/conservatism with two outcomes: accuracy in identifying false news (Study 1, N = 427, RWA-15) and endorsement of conspiracy claims drawn from Romanian electoral discourse (Study 2, N = 170, VSA-6). Adjusted regression models showed that authoritarian submission was the strongest negative correlate of false-news accuracy in Study 1 (β = −0.411, *p* < 0.001), whereas traditionalism was the strongest positive correlate of conspiracy-claim endorsement in Study 2 (β = 0.529, *p* < 0.001). Authoritarian aggression contributed modestly to conspiracy-claim endorsement (β = 0.178, *p* = 0.006) but did not independently predict false-news accuracy (β = −0.075, *p* = 0.130). A non-parametric bootstrap (10,000 resamples) supported the cross-study differences for the traditionalism/conventionalism and submission/conservatism contrasts (both *p* < 0.001), whereas the aggression contrast was not significant. Person-centred analyses in Study 2 were descriptively consistent with the regression results. Because instrument, sample, outcome, and period differed simultaneously, the cross-study contrasts are hypothesis-generating rather than evidence of a causal or construct-level dissociation. Moreover, VSA-6 subdimension analyses should be interpreted as exploratory because the scale was developed primarily to assess overall authoritarianism and the Romanian two-item aggression and conservatism pairs showed weak internal coherence.

## 1. Introduction

The year 2024 was described as the „super-cycle [electoral] year” in modern history, with more than 60 national elections engaging approximately two billion voters worldwide (Asplund et al., 2025). Concurrently, The World Economic Forum’s Global Risks Report (2024) identifies misinformation and disinformation as one of the leading short-term global risks facing the world (World Economic Forum, 2024). The rise of misinformation has transformed susceptibility to false information from a narrow concern in social psychology into a systemic vulnerability of liberal democracies (Lewandowsky et al., 2017; Bennett & Livingston, 2018; Pennycook & Rand, 2021; Mutu, 2024).

In the past decade, authoritarianism has emerged as an important but contested correlate of misinformation-related beliefs and judgments. A growing body of work links right-wing authoritarianism (RWA) to greater acceptance of fake or distorted news (Sindermann et al., 2020; Sinclair et al., 2020; Frischlich et al., 2021), conspiracy belief (Douglas et al., 2017; Frenken et al., 2023), and rejection of scientific evidence (Lewandowsky et al., 2013). At the same time, research in Central and Eastern Europe suggests that associations involving aggregate RWA scores may attenuate or vary after accounting for political orientation, conspiracy mentality, or related ideological variables (Faragó et al., 2020; Szebeni et al., 2021). This raises an underexplored question: do aggression, conventionalism/traditionalism, and submission/conservatism show different independent associations with misinformation-related outcomes? The present study addresses this question in two independent Romanian samples using separate measures of fake-news detection and conspiracy-claim endorsement.

Romania provides a politically salient setting for this question. On 6 December 2024, the Constitutional Court annulled the 2024 presidential electoral process and ordered that it be resumed in full (Constitutional Court of Romania, 2024). Declassified materials and subsequent analyses described irregular online amplification surrounding Călin Georgescu’s campaign, while VIGINUM later documented artificial promotion of content on TikTok and the instrumentalization of influencers (Mutu, 2026; VIGINUM, 2025). Importantly, VIGINUM explicitly stated that its report did not itself characterize a foreign digital interference operation (VIGINUM, 2025). The electoral crisis therefore provides a naturalistic and politically salient context, not a quasi-experiment. The Constitutional Court ruling concerned the electoral process; it did not fact-check each conspiracy statement used in the present study. Accordingly, Study 2 is framed as measuring conspiracy-claim endorsement rather than treating every item as institutionally invalidated misinformation.

### 1.1. Authoritarianism: From Composite Score to Subdimensions

The concept of right-wing authoritarianism (RWA), developed by Altemeyer (1981, 1996) on the foundations of authoritarian personality by Adorno et al. (1950), describes an attitudinal syndrome with three components: authoritarian aggression (sanctioning of norm violators), conventionalism (adherence to traditional norms), and authoritarian submission (deference to legitimate authority). Duckitt et al. (2010) subsequently reconceptualized these dimensions as social attitudes rather than personality traits, renaming them authoritarianism, traditionalism, and conservatism, and operationalizing them in the Authoritarianism–Conservatism–Traditionalism (ACT) scale. Bizumic and Duckitt (2018) later developed an ultra-short six-item version of this instrument, the Very Short Authoritarianism (VSA-6) scale.

The bulk of empirical work, however, has continued to treat authoritarianism as a unitary construct, using either total RWA scores or short-form aggregates without disaggregating by subdimension (e.g., Sindermann et al., 2020; Frenken et al., 2023). This practice obscures potentially important differences. Sinclair et al. (2020), for example, demonstrated that authoritarianism is negatively associated with the updating of beliefs after prediction errors—an effect more plausibly attributable to cognitive rigidity (linked to submission and traditionalism) than to behavioral intolerance (linked to aggression). Likewise, Duckitt and Sibley’s (2010) dual-process model identifies separate motivational substrates for the agonistic and traditionalist components of authoritarianism, implying that they should also have different cognitive consequences.

### 1.2. Two Forms of Misinformation Susceptibility

Contemporary research often treats fake news and conspiracy theories as twin manifestations of a generic “misinformation” phenomenon (Pennycook & Rand, 2021). We argue that this approach obscures fundamental psychological differences between the two processes. Fake news detection can be understood primarily as a judgment task, a cognitive discrimination operation. The respondent evaluates the veracity of a news item based on formal cues (source, tone, factual plausibility) and comparison with prior knowledge (Pennycook & Rand, 2019). This process is well captured by signal detection theory (Wickens, 2002) and we used it in our methodology in order to measure the accuracy of discriminating between true and fake news. Crucially, it is not inherently ideologically charged: even highly committed partisans can correctly classify implausible news, provided that they pause to evaluate it analytically (Pennycook & Rand, 2019, p. 49).

The two outcomes examined here are related but not interchangeable, and it is worth being precise about how they differ. Veracity discernment is a performance measure: participants are shown items of known truth value and their accuracy indexes epistemic vigilance, source evaluation and willingness to engage analytically with a claim (Pennycook & Rand, 2019; Bronstein et al., 2019). Conspiracy belief is an attitudinal measure: it indexes endorsement of a worldview about how power operates, which Imhoff and Bruder (2014) characterise as a generalised political attitude rather than a set of discrete propositions. Nera (2024) has since argued that conspiracy mentality is better understood as a set of descriptive assumptions about the functioning of the social world, which clarifies why it behaves like an ideological orientation rather than like a reasoning deficit.

The distinction matters because the two outcomes need not share antecedents. Evidence on this point is mixed: Bronstein et al. (2019) find that belief in fake news and conspiracy ideation share associations with reduced analytic thinking and dogmatism, whereas Wintterlin (2025) report that both are predicted principally by conspiracy mentality and by the perception that truth is politically constructed, suggesting that generalised institutional distrust may matter more than the formal differences between the two types of content. Faragó et al. (2020), working in an Eastern European context comparable to ours, find conspiracy mentality to be an independent determinant of fake news susceptibility once ideological congruence is accounted for.

We therefore do not treat the two outcomes as interchangeable indicators of a single latent susceptibility. We treat them as distinct dependent variables that may be predicted by different components of the same ideological syndrome—which is the question this paper asks. A limitation follows directly and we state it here rather than in the discussion: because each outcome was measured in a different sample, our design cannot estimate their empirical overlap, and any inference about their distinctness rests on the pattern of predictors rather than on a direct comparison.

This distinction suggests, but does not establish, differentiated predictions. One account holds that submission reflects deference to perceived authority—a disposition to accept information bearing the markers of legitimate sources without further scrutiny—which would implicate it in veracity discernment specifically, through the heuristic route described in credibility-evaluation models (Metzger et al., 2010; Traberg & van der Linden, 2022). A second account holds that traditionalism reflects commitment to an established social order (Duckitt et al., 2010), providing motivational grounds for accepting narratives that attribute social disruption to concealed actors—the process Kunda (1990) termed motivated reasoning and that Kahan (2017) and Van Bavel and Pereira (2018) have developed as identity-protective cognition, in which the function of reasoning is to defend a valued group identity rather than to arrive at an accurate belief.

We emphasise that these are competing interpretations of a predictor pattern, not tested mechanisms. Neither study measured cognitive reflection, need for cognitive closure, epistemic trust, media literacy or identity-protective motivation, and a cross-sectional design could not establish mediation even if it had. We report in the Limitations a partial test of the closure-based account in an independent Romanian sample, which did not support it. Readers should treat the mechanisms proposed here as hypotheses for the crossed, mechanism-instrumented design we describe in Future Directions.

### 1.3. Romania as an Empirical Setting

Romania remains under-represented in the international literature on misinformation and conspiracy belief, despite a political environment in which electoral polarization, nationalist rhetoric, and online information manipulation have become highly salient (Bârgăoanu et al., 2023; Botan et al., 2025; Cucu, 2023). The December 2024 electoral crisis added a distinctive naturalistic setting for the present research: Study 2 was conducted shortly after the annulment, when election-related conspiracy claims remained cognitively and politically salient. These claims were publicly contested in a context of institutional scrutiny of the electoral process, but the court decision should not be interpreted as an item-by-item validation or invalidation of the statements used in our scale (Constitutional Court of Romania, 2024; VIGINUM, 2025).

The use of two complementary authoritarianism instruments (RWA-15 in Study 1 and VSA-6 in Study 2) also permits an exploratory comparison of conceptually corresponding dimensions. This comparison is necessarily limited. The RWA-15 was developed as a short overall RWA measure, and the present three-subdimension scoring is empirically derived; the VSA-6 was likewise designed primarily to assess overall authoritarianism, with its developers explicitly cautioning against treating the three two-item content pairs as validated standalone subscales (Bizumic & Duckitt, 2018; Zakrisson, 2005). We therefore use cross-instrument similarities as conceptual and predictive convergence only, not as evidence of measurement invariance or structural equivalence.

### 1.4. The Present Study

We pursue three research questions and five hypotheses, derived from the theoretical considerations above.

The first set of hypotheses follows from the distinction developed above. Because authoritarian submission reflects deference to authority, it may be associated with poorer veracity judgments when source or authority cues substitute for independent evaluation (H1a). Because traditionalism reflects commitment to established social and moral orders, it may be more strongly associated with endorsement of politically and culturally value-laden conspiracy narratives (H1b). Authoritarian aggression, which concerns sanctioning norm violators, was not expected a priori to contribute independently after the other dimensions were controlled (H1c). These propositions concern predictor patterns; the underlying cognitive and motivational mechanisms were not directly measured.

RQ1. Which authoritarianism dimension—aggression, conventionalism/traditionalism, or submission/conservatism—shows the strongest independent association with the focal outcome in each Romanian sample?

**H1a.**  *Authoritarian submission negatively predicts accuracy in identifying false news after controlling for demographics and the other RWA dimensions.*

**H1b.**  *Conventionalism/traditionalism positively predicts conspiracy belief, emerging as the strongest predictor among the authoritarianism subdimensions.*

**H1c.**  *Authoritarian aggression does not significantly predict either form of susceptibility after controlling for the other subdimensions.*

RQ2. Does the predictive pattern differ between the two outcomes (fake news detection vs. conspiracy belief)?

**H2.**  *Cross-study contrasts will show a larger traditionalism/conventionalism coefficient for conspiracy-claim endorsement than for fake-news detection and a larger submission/conservatism coefficient for fake-news detection than for conspiracy-claim endorsement; no significant cross-study difference is expected for aggression. Because outcome, instrument, sample, and period differ simultaneously, this hypothesis is treated as an exploratory between-study contrast rather than a direct test of outcome-specific mechanisms.*

RQ3. Is there convergence between predictions derived from the two instruments (RWA-15 and VSA-6)?

**H3.**  *Conceptually corresponding dimensions across the two instruments—submission (RWA-15) ↔ conservatism (VSA-6) and conventionalism (RWA-15) ↔ traditionalism (VSA-6)—will show some convergent predictive patterns. Any convergence will be interpreted as exploratory cross-instrument evidence rather than as measurement or structural equivalence.*

The article is structured as follows. Section 2 describes the methodology we employed and the integrated analytical strategy. Section 3 presents our results organized by hypotheses, including a cross-study comparison and confirmatory factor analyses. Section 4 discusses theoretical and applied implications. Section 5 addresses limitations and future avenues for research.

## 2. Materials and Methods

### 2.1. Overview and Design

We employed a two-study design with independent samples to examine associations between authoritarianism dimensions and two distinct outcomes. Study 1 (N = 427) assessed false-news detection accuracy using the Right-Wing Authoritarianism scale (RWA-15; Zakrisson, 2005) and a custom news-evaluation task. Study 2 (N = 170) assessed conspiracy-claim endorsement using the Very Short Authoritarianism scale (VSA-6; Bizumic & Duckitt, 2018), a set of conspiracy statements drawn from Romanian electoral discourse, and selected items from the Popular Epistemically Unwarranted Beliefs Inventory (PEUBI; Huete-Pérez et al., 2022). Both studies were conducted in Romania between 2024 and 2025.

The studies used different authoritarianism instruments by design, allowing an exploratory comparison of conceptually analogous content domains across measurement approaches. Prior research has often related aggregate RWA scores to misinformation-related outcomes (Frischlich et al., 2021; Sinclair et al., 2020), whereas distinct authoritarianism dimensions have more often been examined in research on prejudice and intergroup attitudes (Duckitt & Bizumic, 2013; Dunwoody & Funke, 2016). Because the instruments share no items and because the VSA-6 was developed primarily as an overall RWA measure rather than as three validated two-item subscales (Bizumic & Duckitt, 2018), the present cross-instrument comparisons are treated as exploratory conceptual convergence, not as tests of measurement invariance or structural equivalence. Both studies were conducted with informed consent and approved by the Doctoral Council of Alexandru Ioan Cuza University of Iași, Faculty of Psychology and Educational Sciences. Data and analysis materials are available from the corresponding author subject to the confidentiality restrictions stated in Data Availability Statement.

### 2.2. Study 1: Fake News Detection

#### 2.2.1. Participants

The corrected Study 1 analytic sample comprised 427 Romanian adults (64.2% women, 35.6% men, 0.2% other; mean education level corresponding approximately to undergraduate education, M = 5.31, SD = 1.06 on a 1–7 scale ranging from primary school to doctoral degree; mean income category M = 4.73, SD = 1.79 on a 1–7 scale). Age was assessed in six brackets: 18–25 (25.5%), 26–35 (16.6%), 36–45 (29.7%), 46–55 (17.1%), 56–65 (5.6%), and 65+ (5.4%). Participants were recruited online via social media advertising and snowball sampling between March and June 2024. No financial compensation was offered.

#### 2.2.2. Materials

Right-Wing Authoritarianism Scale (RWA-15). We administered the 15-item RWA scale in its Romanian adaptation, rated on a 9-point scale (1–9; Zakrisson, 2005). For the present exploratory subdimension analyses, scores were computed as means of four items each: aggression (items 1, 11, 13, 15), conventionalism (items 2, 4, 6, 10), and submission (items 3, 5, 7, 9). Items 8, 12, and 14 did not load consistently on these empirically derived factors and were not included in the subdimension scores; the analytic total used here is therefore the mean of the 12 scored items (α = 0.783), while α across all 15 items was 0.738. These subdimension scores should not be interpreted as standard validated RWA-15 subscales. Moreover, all four conventionalism items are reverse-worded, so this factor may partly reflect a wording-method effect rather than a pure content dimension (Kam, 2023; Weijters & Baumgartner, 2012; Zhang et al., 2016); this issue is revisited in the Limitations.

News Evaluation Task. Participants evaluated 16 short news items—8 true and 8 false—selected using the News Weight Coefficient procedure described by Răileanu-Olariu and Oprea (2025). Accuracy on false news (ACC_F) and accuracy on true news (ACC_A) were computed as the proportion correct within each set of eight items. Signal-detection sensitivity (d′) was computed over the same 8 + 8 items using the Snodgrass–Corwin log-linear correction (Snodgrass & Corwin, 1988). The previous version of the manuscript reported k = 12 for these indices; that value was carried over from an earlier analysis and is corrected here.

Demographic variables. Participants reported gender (woman, man, or other), age (six ordinal brackets), highest level of education completed (1–7), monthly personal income (1–7), residential setting (urban/peri-urban/rural), and political preference. For regression analyses, gender was represented by a woman-versus-man dummy contrast (woman = 1, man = 0); the single other response was treated as missing for this binary contrast.

#### 2.2.3. Procedure

Data were collected in an online session lasting approximately 25 min. The order of presentation was the demographic block, RWA-15 (fixed item order), and the news-evaluation task (randomized item order across participants). Attention checks were embedded in the RWA-15 block. The corrected analytic dataset used for the present manuscript contains N = 427 cases after data cleaning; for the main regression models, listwise deletion of cases with missing education or gender yielded N = 420.

### 2.3. Study 2: Conspiracy Belief

#### 2.3.1. Participants

Study 2 enrolled 170 Romanian adults (62.4% women, 37.6% men; education M = 4.28, SD = 1.34; income M = 3.57, SD = 1.89). The age distribution was younger than in Study 1: 18–25 (50.6%), 26–35 (10.0%), 36–45 (8.8%), 46–55 (20.0%), 56–65 (8.8%), and 65+ (1.8%). Recruitment took place during February and March 2025, shortly after the Constitutional Court’s 6 December 2024 annulment of the presidential electoral process. The demographic composition of this convenience sample is treated as a limitation rather than as representative of Romanian social-media users or of the national population.

#### 2.3.2. Materials

Very Short Authoritarianism Scale (VSA-6). We used the six-item VSA developed by Bizumic and Duckitt (2018). The VSA includes one protrait and one contrait item from each of the ACT content domains—authoritarianism/aggression, traditionalism/conventionalism, and conservatism/submission—but it was developed primarily to provide a very short overall RWA score. Bizumic and Duckitt (2018) explicitly cautioned that separate two-item aggression and submission/conservatism scores were not adequately differentiated in their validation studies. Responses in the present study were linearly transformed from the original 1–9 coding to a centred −4 to +4 metric; reverse-keyed items (1, 4, and 5) were recoded before scoring. This linear transformation does not alter correlations or standardized regression coefficients. Consequently, the three two-item component scores used below are exploratory, with their internal coherence reported explicitly.

Conspiracy Statements (CS). We compiled 16 conspiracy statements drawn from Romanian electoral and public discourse and grouped them by content: electoral conspiracy (4 items), historical conspiracy (4 items), religious/spiritual conspiracy (3 items), health-related conspiracy (3 items), and technology-related conspiracy (2 items). Each statement was rated from 1 (completely disagree) to 5 (completely agree). The primary outcome, CS mean, was the mean across all 16 items (α = 0.943). For a descriptive person-centred analysis only, participants were grouped using the empirical thresholds present in the corrected dataset: Skeptics (n = 31, CS mean ≤ 1.0625), Neutrals (n = 98, 1.0625 < CS mean ≤ 2.75), and Believers (n = 41, CS mean > 2.75). Because these categories are derived from the same outcome used in the regression analyses, group comparisons are descriptive and are not treated as independent validation.

Popular Epistemically Unwarranted Beliefs Inventory (PEUBI). As auxiliary convergent and discriminant outcomes, we used a Romanian translation of a selected 17-item subset of the 36-item PEUBI developed by Huete-Pérez et al. (2022). In the corrected dataset, six selected items form the Conspiracy Theories score (PEUBI-CT) and 11 form the Occultism/Pseudoscience score (PEUBI-OP). Items were rated on a 5-point Likert scale and keyed according to the selected-item scoring used in the study. Internal consistency in the present sample was α = 0.857 for PEUBI-CT, α = 0.838 for PEUBI-OP, and α = 0.858 across the 17 selected keyed items. Because the present study used a subset rather than the full validated 36-item PEUBI, these scores are treated as auxiliary measures and not as a separately validated Romanian adaptation of the full instrument.

Auxiliary measures (Status Anxiety, Anomia). To assess competing predictors in exploratory robustness analyses, we administered the five-item Status Anxiety Scale (SAS; α = 0.880; Keshabyan & Day, 2020; Melita et al., 2020) and the six-item Middleton Anomia Scale (MAS; α = 0.564; Middleton, 1963). These variables were used as auxiliary covariates rather than as primary predictors.

#### 2.3.3. Procedure

Data were collected in an online session lasting approximately 30 min. The order of presentation was the demographic block, conspiracy statements (randomized), VSA-6, PEUBI items, SAS, MAS, and a final block including political preference and information-consumption habits. No financial compensation was offered. One case had missing education data and was excluded by listwise deletion from the regression models, yielding N = 169; descriptive statistics use the full N = 170.

### 2.4. Analytic Strategy

All analyses were conducted in Python 3.12 using the libraries pandas, statsmodels, scipy, and semopy. Parallel analyses were performed using IBM SPSS Statistics v26.

The analytic plan proceeded in four steps. First, for each regression model, continuous predictors and the outcome were z-standardised among the cases entering that model. Gender was dummy-coded (1 = woman, 0 = man). Accordingly, coefficients for continuous predictors are standardized β coefficients; the gender coefficient represents the adjusted woman-versus-man difference in outcome standard-deviation units.

Second, we estimated a demographic baseline model (gender, age, education, income) in each study. We then evaluated two expansions of that same baseline: (a) an aggregate-score model adding RWA total in Study 1 or VSA total in Study 2, and (b) an alternative subdimension model in which the three authoritarianism components replaced the global score and were entered simultaneously. ΔR^2^ and F-change statistics for both expansions were therefore computed relative to the demographic baseline; no F-change test is interpreted between the aggregate-score and subdimension models because those models are not nested. Incremental Cohen’s f^2^ for the subdimension model was also computed relative to the demographic baseline (Cohen et al., 2003). Variance inflation factors were inspected for multicollinearity (VIF < 5; Hair et al., 2014).

Third, we tested cross-study differences in the subdimension coefficients. Because lower accuracy denotes greater susceptibility in Study 1 whereas higher conspiracy endorsement denotes greater susceptibility in Study 2, Study 1 coefficients were sign-reversed to create a common directional metric. Standardized coefficients were then compared across the two independent samples using a non-parametric bootstrap with 10,000 independent resamples and percentile confidence intervals. This procedure avoids relying on a normal approximation for the difference between partial regression coefficients, but the resulting contrasts remain between-study comparisons and cannot isolate the effects of outcome, instrument, sample, or period.

Fourth, confirmatory factor analyses (CFA) were estimated separately for the RWA items and the VSA-6 using maximum-likelihood estimation. Model fit was summarized with χ^2^, CFI, TLI, RMSEA, and SRMR, using conventional benchmarks as descriptive guides rather than rigid decision rules (Hu & Bentler, 1999). Because the instruments contain different items and the VSA-6 was not designed for validated standalone two-item subscale scoring (Bizumic & Duckitt, 2018), these analyses evaluate internal factor structure within each instrument; they do not constitute a test of measurement invariance or structural equivalence across instruments.

Finally, as a complementary person-centred description, we conducted one-way ANOVAs in Study 2 comparing the three CS-derived belief profiles on each authoritarianism component, with Tukey HSD post-hoc tests and η^2^ effect-size estimates. Because the profiles were created from the outcome itself, these comparisons are illustrative rather than independent replications of the variable-centred analyses.

### 2.5. Use of Generative AI

Generative AI tools were used during manuscript preparation. Anthropic Claude (Sonnet 4.6) assisted with translation and academic-English refinement. OpenAI ChatGPT (GPT-5.6 Sol, accessed August 2026) was used to assist with statistical-consistency checking against the corrected datasets, bibliographic verification against primary sources, and language/editorial revision. AI systems were not treated as authors or as sources of empirical evidence. All AI-assisted outputs were checked against the underlying datasets and cited sources by the authors, who take full responsibility for the analyses, interpretations, citations, and final manuscript.

## 3. Results

### 3.1. Descriptive Statistics and Reliability

Table 1 reports descriptive statistics for the main predictor and outcome variables. The two convenience samples differed demographically, particularly in age composition; age, education, income, and gender were therefore retained as common demographic covariates in the regression models.

Internal consistency was acceptable for the 12 scored RWA items (α = 0.783; α = 0.738 across all 15 items) and excellent for the 16-item conspiracy-statements score (α = 0.943). For the corrected VSA-6, α = 0.471, well below the α = 0.76 reported in one of the original validation samples (Bizumic & Duckitt, 2018). The two traditionalism items showed a moderate inter-item correlation (r = 0.467), whereas the aggression (r = 0.028) and conservatism (r = −0.049) pairs showed essentially no internal coherence. This pattern reinforces the need to treat VSA-6 component analyses as exploratory. The selected PEUBI scores showed good internal consistency in the present sample (PEUBI-CT α = 0.857; PEUBI-OP α = 0.838).

### 3.2. Bivariate Relationships

**Figure 1:**
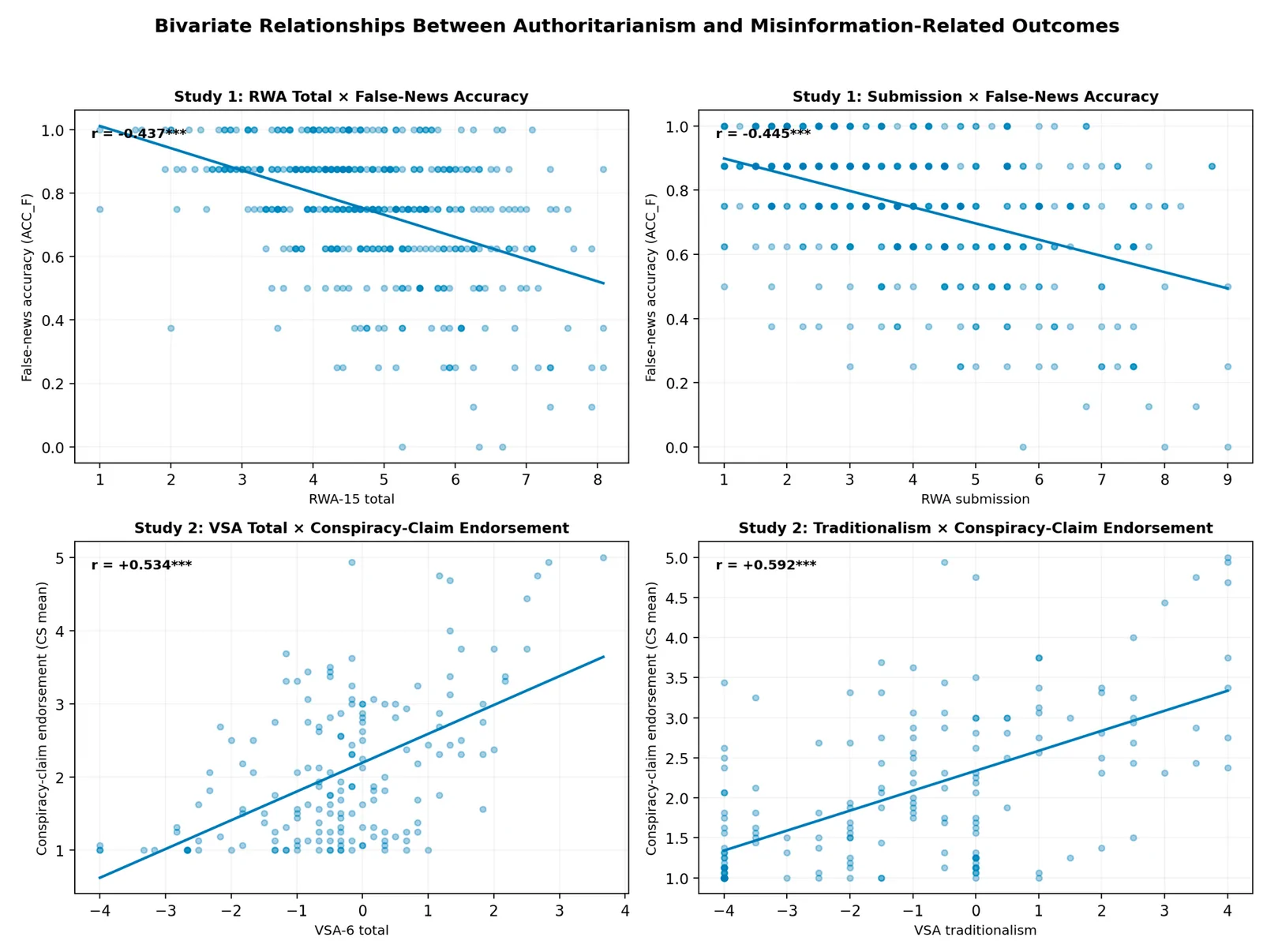
Bivariate relationships between authoritarianism scores and the focal outcomes in Study 1 (**top** panels) and Study 2 (**bottom** panels). The four correlations displayed are significant at *** *p* < 0.001.

Figure 1 illustrates Pearson correlations between authoritarianism scores and the primary outcomes in each study. In Study 1, authoritarian submission showed the strongest negative association with accuracy on false news (r = −0.445, *p* < 0.001), exceeding the 12-item RWA total (r = −0.437). Aggression correlated −0.286 with false-news accuracy but was unrelated to true-news accuracy (r = −0.017, *p* = 0.733). Conventionalism correlated similarly with both outcomes (r = −0.241 and r = −0.238), a pattern consistent with a general response tendency rather than with content-specific discrimination.

In Study 2, using the corrected scores, traditionalism was the strongest bivariate correlate of conspiracy-claim endorsement (r = 0.592, *p* < 0.001), followed by the VSA total (r = 0.534, *p* < 0.001) and aggression (r = 0.255, *p* < 0.001); conservatism showed a weak association (r = 0.181, *p* = 0.018). Associations with PEUBI-CT followed the same general ordering (traditionalism r = 0.383; aggression r = 0.294; conservatism r = 0.039), whereas associations with PEUBI-OP were weaker (traditionalism r = 0.258; aggression r = 0.149; conservatism r = −0.006). Thus, the auxiliary outcomes indicate stronger authoritarianism associations with conspiracy content than with occult/pseudoscientific content, but they do not support complete domain specificity.

### 3.3. Hierarchical Regression Models

#### 3.3.1. Study 1: Predicting Fake News Accuracy

Table 2 reports the regression models for Study 1, with z-standardised false-news accuracy (ACC_F) as the outcome. Listwise deletion left N = 420 of the 427 participants: six cases had missing education data and one additional case lacked a usable binary gender value. The previously reported N = 421 resulted from retaining that latter case in a binary contrast and has been corrected.

In the alternative subdimension model, authoritarian submission was the only significant authoritarianism component predicting false-news accuracy (β = −0.411, SE = 0.053, t = −7.78, *p* < 0.001, 95% CI [−0.514, −0.307]). Aggression (β = −0.075, *p* = 0.130) and conventionalism (β = −0.071, *p* = 0.117) were not independently significant after adjustment for demographics and the other dimensions. Thus, their zero-order associations with ACC_F did not remain statistically independent in the multivariable model. Multicollinearity was low (all VIFs < 1.55).

#### 3.3.2. Study 2: Predicting Conspiracy Belief

Table 3 reports the corresponding Study 2 models with z-standardised mean conspiracy-claim endorsement as the outcome and the corrected VSA scores as predictors. The demographic baseline accounted for R^2^ = 0.116, F(4, 164) = 5.37, *p* < 0.001. Adding VSA total increased R^2^ to 0.347 (ΔR^2^ = 0.232, ΔF(1, 163) = 57.82, *p* < 0.001; VSA total β = 0.506, *p* < 0.001). The alternative three-component model reached R^2^ = 0.417 relative to the same demographic baseline (ΔR^2^ = 0.301, ΔF(3, 161) = 27.74, *p* < 0.001; incremental f^2^ = 0.517).

In the alternative component model, traditionalism was the strongest predictor of conspiracy-claim endorsement (β = 0.529, SE = 0.066, t = 7.99, *p* < 0.001, 95% CI [0.399, 0.660]). Authoritarian aggression made a smaller but significant contribution (β = 0.178, SE = 0.064, t = 2.77, *p* = 0.006, 95% CI [0.051, 0.305]), whereas conservatism/submission was not significant (β = 0.021, *p* = 0.749). Among the demographic covariates, education was negatively associated with conspiracy-claim endorsement (β = −0.185, *p* = 0.009); age, gender, and income were not significant. These values supersede the coefficients reported in the earlier preprint/manuscript version that used incorrectly scored VSA items.

### 3.4. Cross-Study Comparison of Effect Sizes

**Figure 2:**
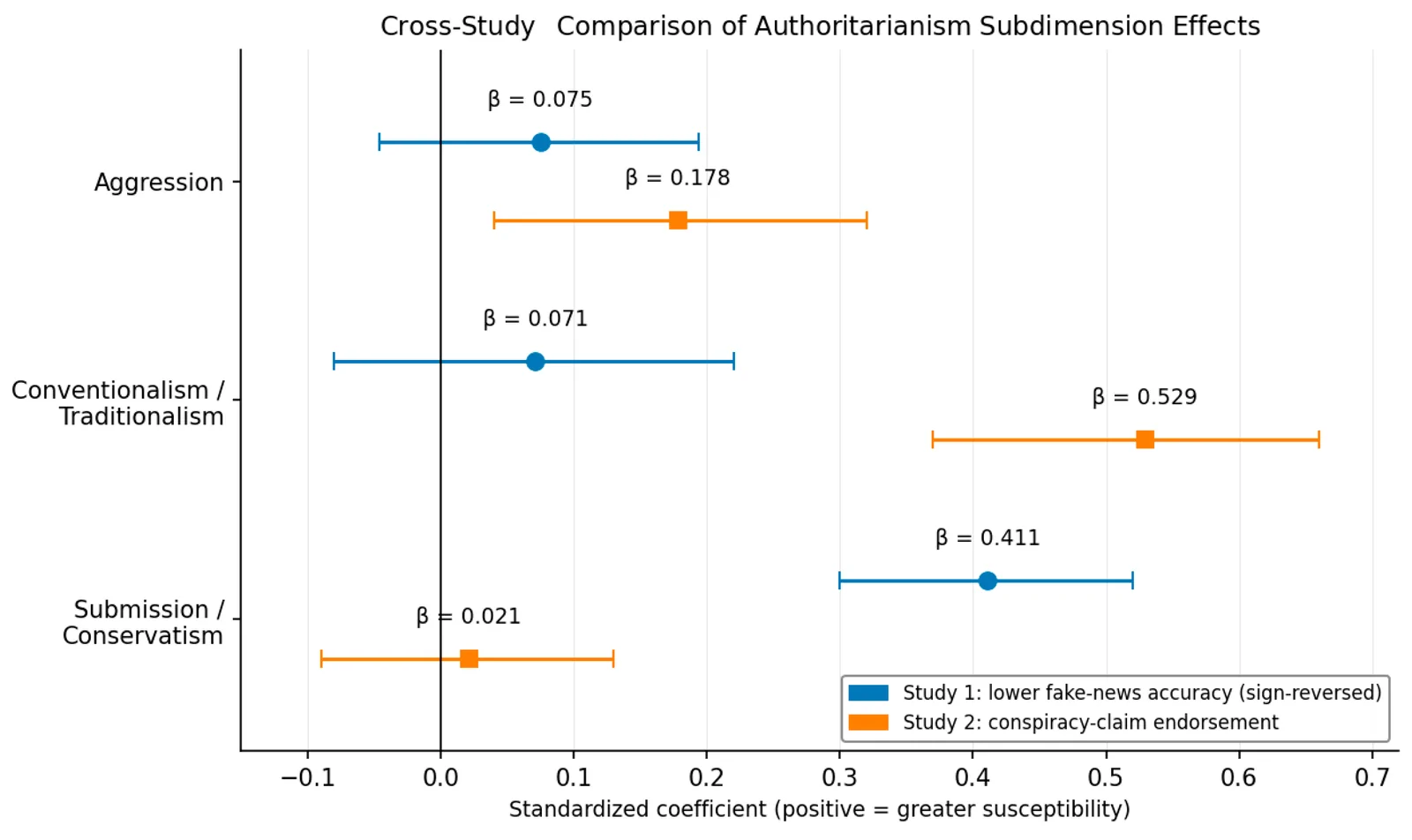
Forest plot of regression coefficients for authoritarianism components predicting the focal outcomes. Study 1 coefficients are sign-reversed for directional comparability. Bars represent 95% confidence intervals.

Figure 2 displays coefficients from the two alternative subdimension/component models. For directional comparability, Study 1 coefficients are sign-reversed so that positive values indicate greater susceptibility (lower accuracy in Study 1 and higher conspiracy endorsement in Study 2). The plot shows a differentiated cross-study pattern: submission is the largest Study 1 coefficient, traditionalism is the largest Study 2 coefficient, and aggression is non-significant in Study 1 but significant in Study 2. Because these comparisons cross instruments, samples, outcomes, and periods, the figure is descriptive rather than evidence of a construct-level dissociation.

Table 4 reports the bootstrap cross-study contrasts. Fisher’s r-to-z transformation, used in an earlier version, is not appropriate for comparing partial regression coefficients estimated in independent samples with different measures and outcomes; it was therefore replaced with a non-parametric bootstrap (10,000 resamples, percentile intervals). The traditionalism/conventionalism contrast was positive (Δβ = 0.458, 95% CI [0.281, 0.621], *p* < 0.001), whereas the submission/conservatism contrast was negative (Δβ = −0.390, 95% CI [−0.545, −0.232], *p* < 0.001). The aggression contrast was not significant (Δβ = 0.103, 95% CI [−0.039, 0.241], two-sided *p* = 0.165). These tests quantify cross-study differences but cannot attribute them specifically to outcome type because instrument, sample, and period also vary.

### 3.5. Person-Centered Analysis: Conspiracy Belief Profiles

**Figure 3:**
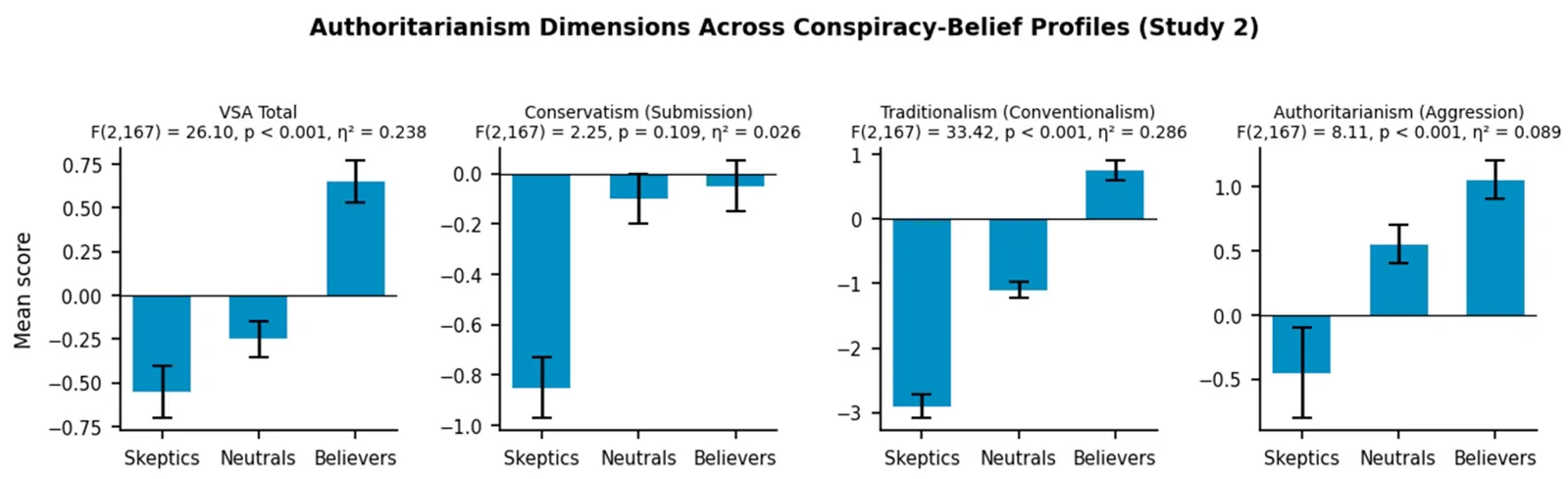
Authoritarianism subdimensions across conspiracy belief profiles in Study 2 (Skeptics, n = 31; Neutrals, n = 98; Believers, n = 41). Error bars represent ±1 SE. Note the contrasting patterns: VSA Total and Traditionalism increase monotonically with belief; Aggression shows a modest but significant pattern (η^2^ = 0.089), while Conservatism does not differentiate groups.

To complement the variable-centered analyses, we examined whether authoritarianism subdimensions differ among the three conspiracy belief profiles in Study 2 (Skeptics, Neutrals, Believers) (classified using empirically derived thresholds as described in Section 2.3.2). Figure 3 displays the group means and the corresponding one-way ANOVA results with η^2^ effect sizes.

Using the corrected scores, one-way ANOVA showed substantial differences among groups for the VSA total (F(2, 167) = 26.10, *p* < 0.001, η^2^ = 0.238), traditionalism (F(2, 167) = 33.42, *p* < 0.001, η^2^ = 0.286) and aggression (F(2, 167) = 8.11, *p* < 0.001, η^2^ = 0.089), but not for conservatism (F(2, 167) = 2.25, *p* = 0.109, η^2^ = 0.026). Because the grouping variable is derived from the outcome itself, these analyses are descriptive and are reported to illustrate the variable-centred findings, not as independent confirmation of them.

### 3.6. Confirmatory Factor Analysis

CFA models were estimated separately to examine the internal factor structure of the empirically scored RWA items in Study 1 and the corrected VSA-6 items in Study 2. Because the instruments contain different items and have different scale-development purposes, these analyses do not test cross-instrument structural equivalence.

For the 12 RWA items used in the exploratory subdimension scores, the three-factor model showed mixed fit: χ^2^(51) = 163.18, CFI = 0.905, TLI = 0.877, RMSEA = 0.072, SRMR = 0.063. CFI, RMSEA, and SRMR were within commonly used approximate-fit ranges, whereas TLI was below the prespecified 0.90 benchmark (Hu & Bentler, 1999). The three-factor model nevertheless fitted substantially better than a one-factor model, χ^2^(54) = 409.31, CFI = 0.700, TLI = 0.633, RMSEA = 0.124, SRMR = 0.100; Δχ^2^(3) = 246.13, *p* < 0.001. Latent correlations were 0.711 for submission–aggression, 0.494 for submission–conventionalism, and 0.274 for aggression–conventionalism. These results provide some support for empirical separability in this sample, but they should be interpreted alongside the post-hoc subscale construction and the confounding of the conventionalism score with reverse wording.

For the corrected VSA-6, the three-factor model is statistically overidentified (df = 6) but psychometrically fragile because each factor has only two indicators. Fit was poor: χ^2^(6) = 24.05, CFI = 0.788, TLI = 0.471, RMSEA = 0.133, SRMR = 0.070. A one-factor model also fitted poorly, χ^2^(9) = 33.74, CFI = 0.710, TLI = 0.517, RMSEA = 0.128, SRMR = 0.087. Together with the very low inter-item correlations for the aggression and conservatism pairs, these findings do not validate three standalone Romanian VSA-6 subscales. This interpretation is also consistent with Bizumic and Duckitt’s (2018) explicit conclusion that the VSA-6 was designed to assess overall RWA and that separate two-item aggression and submission/conservatism scores were not adequately differentiable.

Accordingly, the CFA results do not establish structural equivalence across the RWA-15 and VSA-6. Cross-instrument comparisons elsewhere in the manuscript refer only to conceptual correspondence between content domains. Study 2 component-level findings, especially those involving aggression and conservatism/submission, should be treated as exploratory pending replication with a longer multidimensional authoritarianism measure.

### 3.7. Robustness Checks

Five robustness checks were performed. First, we replicated the Study 1 regression using signal-detection sensitivity (d′) as the outcome. The pattern was broadly consistent with that obtained for ACC_F: authoritarian submission remained the strongest negative predictor (β = −0.371, *p* < 0.001), while conventionalism also showed an independent negative association (β = −0.198, *p* < 0.001); authoritarian aggression was not significant (β = −0.018, *p* = 0.713). Thus, the association between authoritarian submission and poorer fake-news discernment was not limited to the proportion-correct measure and was also observed for a signal-detection index that accounts for response tendencies.

Second, we replicated the Study 2 regression using PEUBI-CT as an alternative measure of conspiracy belief rather than the custom conspiracy-statements (CS) scale. Traditionalism again emerged as the strongest authoritarian predictor (β = 0.305, *p* < 0.001), followed by authoritarian aggression (β = 0.268, *p* < 0.001), whereas conservatism/submission was not significantly associated with PEUBI-CT (β = −0.101, *p* = 0.175). This pattern was therefore broadly consistent with the corrected CS model, in which traditionalism was also the dominant predictor and aggression made a smaller but statistically significant independent contribution, whereas conservatism/submission did not contribute significantly. The convergence across two differently constituted measures of conspiracy belief strengthens the evidence that the Study 2 associations are not dependent solely on the content of the custom CS measure.

Third, we examined PEUBI-OP, representing occult and pseudoscientific beliefs, as a discriminant outcome. In the adjusted regression model, traditionalism remained significantly associated with PEUBI-OP (β = 0.240, *p* = 0.004), whereas authoritarian aggression (β = 0.143, *p* = 0.075) and conservatism/submission (β = −0.091, *p* = 0.264) were not statistically significant. Accordingly, the results do not support a fully specific association between authoritarianism and conspiracy belief. Rather, they indicate that the authoritarianism–conspiracy association was substantially stronger and more multidimensional than the corresponding association with occult or pseudoscientific beliefs.

Fourth, the principal Study 2 findings remained stable when residence, anomie, status anxiety, and the three personal-achievement factors were added as covariates, with traditionalism remaining the strongest predictor (βs = 0.516–0.533, all *p*s < 0.001). Fifth, the principal Study 1 result was likewise robust to the inclusion of political orientation, self-efficacy, personal achievement, and information-seeking behaviour, with authoritarian submission remaining strongly associated with poorer fake-news detection (βs = −0.392 to −0.412, all *p*s < 0.001). As an additional inferential sensitivity check, HC3 heteroskedasticity-consistent standard errors preserved the same substantive significance pattern: in Study 1 submission remained significant (*p* < 0.001), whereas aggression (*p* = 0.099) and conventionalism (*p* = 0.182) did not; in Study 2 traditionalism (*p* < 0.001) and aggression (*p* = 0.003) remained significant, whereas conservatism did not (*p* = 0.712).

#### Summary of Findings

To summarize, our analyses yielded five main findings:

(1) Authoritarian submission emerged as the strongest predictor of poorer fake-news detection in Study 1 (β = −0.411, *p* < 0.001), whereas traditionalism emerged as the strongest predictor of conspiracy-claim endorsement in Study 2 (β = 0.529, *p* < 0.001). Both associations remained significant after adjustment for demographic covariates and the other authoritarianism dimensions.
(2) Authoritarian aggression showed an outcome-specific pattern. It did not independently predict fake-news detection once the other authoritarianism dimensions were controlled in Study 1 (β = −0.075, *p* = 0.130), but it made a smaller, statistically significant contribution to conspiracy-claim endorsement in Study 2 (β = 0.178, *p* = 0.006). Thus, the corrected analyses do not support the stronger interpretation that authoritarian aggression is unrelated to both outcomes.
(3) The differences between the corresponding regression coefficients across the two studies were supported by bootstrap resampling for the traditionalism/conventionalism and submission/conservatism contrasts (both *p*s < 0.001), whereas the aggression contrast was not statistically significant. These cross-study differences should nevertheless be interpreted cautiously because instrument, outcome, sample, and historical context varied simultaneously between the two studies. Accordingly, they are better regarded as hypothesis-generating evidence for differential associations rather than as demonstrating a causal or within-sample dissociation between authoritarianism dimensions.
(4) The person-centered analyses were broadly consistent with the variable-centered results. Conspiracy-belief profiles differed substantially in traditionalism and, to a lesser extent, authoritarian aggression, whereas conservatism/submission did not significantly differentiate the three groups. Traditionalism showed the largest between-group differentiation, particularly between the Believer group and the Skeptic and Neutral groups, reinforcing its central association with conspiracy-claim endorsement in Study 2.
(5) Cross-instrument convergence (H3) received only partial support. The two instruments showed some conceptually analogous patterns, but the findings do not establish measurement or structural equivalence between their respective dimensions. Most notably, submission was the dominant authoritarianism predictor of fake-news detection in Study 1 (β = −0.411, *p* < 0.001), whereas the theoretically corresponding conservatism/submission dimension of the VSA-6 was unrelated to conspiracy-claim endorsement in Study 2 (β = 0.021, *p* = 0.749). Conversely, traditionalism was the dominant predictor in Study 2 (β = 0.529, *p* < 0.001), while conventionalism in Study 1 did not independently predict fake-news detection after the other authoritarianism dimensions were controlled. These asymmetries may reflect differences in instrument operationalization, psychometric properties, outcome characteristics, sample composition, or the distinct political contexts captured by the two studies. They should therefore not be interpreted as evidence that the nominally corresponding subscales are functionally interchangeable across instruments.

The implications of these findings, particularly for the development of more precisely targeted prebunking interventions, are discussed in the following section.

## 4. Discussion

Across two independent Romanian samples and two complementary authoritarianism instruments, we observed differentiated patterns of association between authoritarianism dimensions and susceptibility to misinformation-related outcomes. In Study 1, authoritarian submission emerged as the dominant predictor of poorer fake-news detection (β = −0.411, *p* < 0.001), whereas in Study 2, traditionalism emerged as the dominant predictor of conspiracy-claim endorsement (β = 0.529, *p* < 0.001). Authoritarian aggression showed a more outcome-contingent pattern: it did not independently predict fake-news detection after controlling for demographics and the remaining authoritarianism dimensions in Study 1 (β = −0.075, *p* = 0.130), but made a smaller yet statistically significant contribution to conspiracy-claim endorsement in Study 2 (β = 0.178, *p* = 0.006). Bootstrap resampling supported the cross-study differences for the traditionalism/conventionalism and submission/conservatism contrasts (both *p*s < 0.001), whereas the corresponding difference for aggression was not statistically significant. Person-centered analyses in Study 2 were broadly consistent with the variable-centered findings, with conspiracy-belief profiles differing most strongly on traditionalism and, to a lesser extent, authoritarian aggression, whereas conservatism/submission did not significantly differentiate the groups. Because the two studies differed simultaneously in authoritarianism instrument, outcome measure, sample composition, and political context, these findings should not be interpreted as demonstrating a definitive psychological dissociation or structural equivalence between corresponding authoritarianism dimensions. Rather, they provide convergent, hypothesis-generating evidence that different components of authoritarianism may show distinct patterns of association with fake-news detection and conspiracy-claim endorsement. These results refine current understanding of how authoritarianism relates to vulnerability to misinformation in at least four ways.

### 4.1. The Composite Score Conceals Real Differences

The first contribution concerns measurement practice. Research on authoritarianism and misinformation has often relied on aggregate RWA scores or short-form totals rather than on simultaneous comparisons of distinct authoritarianism dimensions (Frischlich et al., 2021; Sindermann et al., 2020; Szebeni et al., 2021). Our findings show why aggregate and component-level results can differ. In Study 1, the 12-item analytic RWA total was negatively associated with false-news accuracy in the aggregate-score model, whereas the alternative component model identified submission as the dominant independent correlate (β = −0.411, *p* < 0.001); aggression (β = −0.075, *p* = 0.130) and conventionalism (β = −0.071, *p* = 0.117) were not independently significant for ACC_F, although conventionalism did predict d′ in the robustness analysis. In Study 2, VSA total was moderately to strongly correlated with conspiracy-claim endorsement (r = 0.534, *p* < 0.001), while the adjusted component model identified traditionalism as the strongest correlate (β = 0.529, *p* < 0.001), followed by aggression (β = 0.178, *p* = 0.006); conservatism/submission was not significant (β = 0.021, *p* = 0.749). These results suggest that aggregate scores can conceal heterogeneous associations among content domains. However, component-level analyses are informative only when the dimensions are measured adequately; given the VSA-6’s development as an overall RWA measure and the weak coherence of two Romanian item pairs, the Study 2 component findings are exploratory rather than evidence for validated standalone VSA subscales (Bizumic & Duckitt, 2018). Future research should therefore combine dimensional hypotheses with instruments specifically designed and validated for dimensional scoring.

### 4.2. Two Routes to Misinformation Susceptibility

Our second contribution concerns a possible distinction between cognitive and ideological routes to misinformation-related outcomes, but the present data do not directly measure either mechanism. Research on fake-news discernment has repeatedly linked better performance to analytic reasoning and attention to accuracy (Pennycook & Rand, 2019, 2021). The strong Study 1 association between submission and poorer false-news accuracy is compatible with the hypothesis that deference to perceived authority or source cues may reduce independent verification in some contexts, but neither epistemic trust nor source-reliance mechanisms were measured here. The result should therefore be interpreted as an association requiring mechanism-specific follow-up rather than as evidence of a demonstrated “cognitive route.”

Study 2 showed a different predictor pattern. Traditionalism was the strongest correlate of conspiracy-claim endorsement (β = 0.529, *p* < 0.001), and aggression made a smaller independent contribution (β = 0.178, *p* = 0.006), whereas conservatism/submission did not. Because the conspiracy items were embedded in a politically salient electoral context, this pattern is consistent with accounts emphasizing ideological motivation, identity, and worldview congruence in conspiracy belief (Douglas et al., 2017; Kunda, 1990; Uscinski et al., 2022; Van Bavel & Pereira, 2018). It does not, however, establish that traditionalism causes conspiracy belief or that ideology is the mechanism responsible for the association.

The bootstrap comparison reinforces the existence of different cross-study coefficient patterns: submission/conservatism was larger on the common susceptibility metric in Study 1 than in Study 2 (Δβ = −0.390, 95% CI [−0.545, −0.232], *p* < 0.001), whereas traditionalism/conventionalism was larger in Study 2 (Δβ = 0.458, 95% CI [0.281, 0.621], *p* < 0.001). The aggression difference was not significant. These contrasts are consistent with the theoretical distinction proposed in the Introduction, but they are not a direct test of outcome-specific mechanisms because outcome measure, authoritarianism instrument, sample composition, and collection period changed together. Replication within the same sample using the same multidimensional authoritarianism instrument and both outcomes is required before a psychological dissociation can be claimed.

### 4.3. The Outcome-Specific Role of Authoritarian Aggression

A third observation concerns the limited role of authoritarian aggression. In Study 1, aggression did not predict fake news detection once submission and conventionalism were controlled, despite a substantial zero-order correlation (r = −0.286)—a pattern consistent with its shared variance with submission (latent r = 0.711). In Study 2, by contrast, aggression made a modest but reliable contribution to conspiracy belief (β = +0.178, *p* = 0.006) and distinguished conspiracy-belief profiles (η^2^ = 0.089). Aggression therefore appears to be outcome-specific rather than globally irrelevant, and the earlier characterisation of it as “surprisingly irrelevant” is withdrawn.

This pattern is compatible with multidimensional accounts of authoritarianism in which agonistic and norm-preserving content domains need not have identical correlates (Duckitt & Sibley, 2010; Duckitt et al., 2010; Passini, 2017). In the present data, aggression showed different independent associations across the two studies, but the design cannot establish functional separability because the studies used different instruments, outcomes, and samples. The result is therefore best treated as an outcome- and context-contingent association to be tested directly in a common design.

### 4.4. Implications for Prebunking and Media Literacy

Affective pathways not examined here. Our account has been framed in cognitive and ideological terms, and this is a limitation of the framing rather than a claim about the phenomenon. A substantial body of work establishes that the acceptance and diffusion of misinformation are shaped by affect as much as by reasoning. Brady et al. (2017) showed that moral-emotional language increases the diffusion of political content within ideological networks, indicating that emotionally charged claims can bypass deliberative evaluation. Vosoughi et al. (2018) found that false news spreads further and faster than true news, and attributed this in part to the novelty and emotional intensity of the false content. More recently, work on discrete emotions within motivated-reasoning frameworks has identified schadenfreude as a socially consequential affective state: Cecconi et al. (2020) show that expressions of malicious joy in social media interaction are bound up with intergroup bias and with the selective processing of information about disliked outgroups.

This is directly relevant to the Romanian case. The conspiracy statements sampled in Study 2 were not neutral propositions; they circulated within a campaign characterised by strong intergroup framing, and several of them targeted identifiable outgroups. It is plausible that affective reactions to those targets contributed to their endorsement independently of, or in interaction with, the authoritarian dispositions we measured. Because no affective variable was administered in either study, we cannot evaluate this, and we identify it as the most significant unexamined dimension of our model. Future work should measure moral outrage, intergroup schadenfreude and threat perception alongside authoritarianism, on the expectation that emotions interact with ideological predispositions rather than merely accompanying them.

The differentiated component pattern also raises applied questions for misinformation interventions. Prebunking and inoculation approaches can reduce susceptibility to common manipulation techniques (Lewandowsky & van der Linden, 2021; Roozenbeek & van der Linden, 2019), but the present studies did not test any intervention. Accordingly, our results do not demonstrate that a one-size-fits-all intervention is inferior, nor do they establish that authoritarianism-based tailoring would improve outcomes. They instead generate a testable hypothesis that intervention effects may vary with motivational and authority-related dispositions.

Profile-tailored prebunking should therefore be treated as a future experimental question rather than as a recommendation derived from these correlational data. Source-evaluation and lateral-reading protocols remain broadly applicable tools for evaluating online information, while reviews of misinformation and conspiracy interventions emphasize the importance of reasoning, source evaluation, and carefully designed corrective or inoculation strategies (Ecker et al., 2022; Lewandowsky & van der Linden, 2021; O’Mahony et al., 2023). A randomized design crossing intervention type with independently measured authoritarianism dimensions would be required to test whether any specific intervention works better for high-submission or high-traditionalism participants.

Intergroup and affective content may provide an additional moderation pathway. Several Study 2 statements involved politically or socially identifiable groups, and prior research shows that moral-emotional language and intergroup affect can influence the diffusion and processing of political information (Brady et al., 2017; Cecconi et al., 2020). Because neither study measured affective responses to the individual items, the present data cannot determine whether such processes contributed to endorsement. Future intervention studies should therefore manipulate both informational technique and social-affective content rather than assuming that cognitive correction alone is sufficient.

### 4.5. The Romanian Context and Generalizability

The Romanian Study 2 sample was collected in an unusually salient political context following the December 2024 electoral crisis. Election-related conspiracy claims remained prominent in public discourse while the electoral process itself was under intense institutional and media scrutiny (Botan et al., 2025; Constitutional Court of Romania, 2024; Mutu, 2026; Soare et al., 2025). The strong association between traditionalism and conspiracy-claim endorsement is compatible with analyses emphasizing traditional values, national sovereignty, religious identity, and anti-globalist rhetoric in that electoral environment (Botan et al., 2025; Cistelecan et al., 2025; Soare et al., 2025). Because the present study is observational, however, it cannot show that the political mobilization produced the individual-level association.

Generalization beyond Romania remains an open empirical question. The present cross-study pattern may depend on the political content of the items, the instruments used, and the demographic composition of the samples. Cross-national replication with the same multidimensional authoritarianism measure, matched sampling procedures, and both outcome types administered within each sample is needed to determine whether similar coefficient patterns recur across political contexts.

## 5. Limitations and Future Directions

Several limitations of the present study should be acknowledged, each of which suggests directions for future research.

### 5.1. Cross-Sectional Design

Both studies employ cross-sectional designs that do not permit causal inference. The predictive associations reported above are consistent with our theoretical framework, but they cannot rule out reverse causation (e.g., regularly accepting conspiracy claims may itself strengthen traditionalist self-identification) or shared antecedent causes (e.g., early socialization, personality traits such as openness to experience). Longitudinal designs measuring authoritarianism and misinformation susceptibility at multiple time points would clarify the directionality of these associations. Even more informative would be intervention studies that experimentally manipulate the salience of submission or traditionalism (e.g., through priming) and measure downstream effects on misinformation judgments.

### 5.2. Sample Limitations

Both samples were recruited through online platforms, social-media advertising, and convenience/snowball procedures. Such non-probability recruitment limits population representativeness and may produce selection effects associated with age, education, political interest, and digital engagement. Study 2 in particular contained a high proportion of participants aged 18–25 (50.6%). Although demographic covariates were included in the regression models, statistical adjustment does not make a convenience sample representative of the Romanian population, and the direction or magnitude of any selection bias cannot be inferred from the present data. Future studies should use more demographically balanced or probability-based sampling where feasible.

### 5.3. Measurement Considerations for the VSA-6

The VSA-6 used in Study 2 showed low internal consistency after correction (α = 0.471), compared with α values above 0.70 in the original validation samples (Bizumic & Duckitt, 2018). The traditionalism pair showed moderate coherence (r = 0.467), whereas the aggression (r = 0.028) and conservatism (r = −0.049) pairs did not. This is not merely a sample-specific inconvenience: Bizumic and Duckitt (2018) explicitly designed the VSA-6 to assess overall RWA and concluded that separate two-item aggression and submission/conservatism scores were not sufficiently differentiable for routine subscale use. The poor CFA fit in the present Romanian sample reinforces that caution. Accordingly, the Study 2 component analyses are exploratory, the null conservatism result should not be interpreted as evidence that the underlying construct is irrelevant, and the significant aggression result also requires replication with a scale designed for multidimensional scoring. A longer measure such as the 36-item ACT scale (Duckitt et al., 2010) or another validated multidimensional RWA instrument is needed for confirmatory tests.

### 5.4. Domain Specificity of Outcomes

The false-news items in Study 1 and conspiracy statements in Study 2 covered multiple domains but were not exhaustive, and the Study 2 statements were especially tied to a salient electoral context. Associations may therefore vary across health, environmental, technological, or less politically charged conspiracy content. The selected PEUBI-CT items provided a useful auxiliary outcome and reproduced the central traditionalism-plus-aggression pattern, but the present study used only a 17-item subset of the original 36-item PEUBI and did not validate a full Romanian adaptation (Huete-Pérez et al., 2022). Likewise, the relation between submission and fake-news detection may depend on source cues, content congruence, and whether authority markers are present. Domain-stratified and source-cue manipulations would provide a stronger test.

### 5.5. Limited Mechanism Tests

We have proposed cognitive and ideological interpretations of the contrast between submission and traditionalism, but neither mechanism was measured directly. The studies did not include cognitive reflection, need for cognitive closure, epistemic trust, media literacy, institutional trust, or identity-protective motivation as mechanism variables. The proposed routes therefore remain theoretical interpretations of predictor patterns rather than tested mediators. Mechanism claims should be evaluated in new samples using direct measures and, ideally, longitudinal or experimental designs. Numerical results from other datasets are not used here as evidence because those data were not part of the two studies analysed in this manuscript.

### 5.6. Directions for Future Research

Four directions for future research follow from these limitations. First, longitudinal designs measuring authoritarianism dimensions and misinformation-related outcomes at multiple time points would clarify temporal ordering. Second, cross-national replication should administer the same multidimensional authoritarianism instrument and both outcome types within each sample, allowing direct within-sample tests of whether predictor patterns differ by outcome. Third, randomized intervention studies should test—rather than assume—whether prebunking or source-evaluation strategies interact with authoritarianism dimensions. Fourth, replication should use longer, psychometrically stronger multidimensional measures, such as the 36-item ACT scale (Duckitt et al., 2010) or Funke’s (2005) multidimensional RWA measure, so that subdimension-level findings can be separated from the limitations of ultra-short or empirically constructed component scores.

### 5.7. Conclusions

The two Romanian studies show that aggregate authoritarianism scores can conceal different component-level associations with misinformation-related outcomes. In Study 1, submission was the strongest independent correlate of poorer false-news detection; in Study 2, traditionalism was the strongest correlate of conspiracy-claim endorsement, with aggression contributing more modestly. These cross-study differences survived bootstrap resampling for the traditionalism/conventionalism and submission/conservatism contrasts, but they cannot be attributed uniquely to outcome type because the studies also differed in instrument, sample, and period. Moreover, the VSA-6 component findings are exploratory given the instrument’s intended use as an overall RWA measure and the weak coherence of two Romanian item pairs. The results therefore support a cautious, hypothesis-generating account of heterogeneous authoritarianism associations rather than a demonstrated causal dissociation between psychological routes. Confirmatory tests require common instruments, common samples, direct mechanism measures, and experimental or longitudinal designs.

## Figures and Tables

**Table 1:** Descriptive statistics for key variables in Study 1 and Study 2.

Variable	*M*	*SD*	Min	Max	*α*	*k* Items
Study 1 (N = 427)						
RWA-15 Total	4.79	1.31	1.00	8.08	0.783	12
Aggression (F1)	6.36	1.71	1.00	9.00	—	4
Conventionalism (F2)	3.99	1.69	1.00	9.00	—	4
Submission (F3)	4.01	1.84	1.00	9.00	—	4
Accuracy false news (ACC_F)	0.75	0.21	0.00	1.00	—	8
Accuracy true news (ACC_A)	0.62	0.20	0.00	1.00	—	8
d’ (sensitivity)	0.98	0.79	—	—	—	—
Study 2 (N = 170)						
VSA-6 Total	−0.32	1.35	−4.00	3.67	0.471	6
Authoritarianism (Aggression)	0.59	1.81	−4.00	4.00	—	2
Traditionalism (Conventionalism)	−1.08	2.37	−4.00	4.00	—	2
Conservatism (Submission)	−0.48	1.69	−4.00	4.00	—	2
Conspiracy belief (CS mean)	2.07	1.00	1.00	5.00	0.943	16
PEUBI-Conspiracy Theories	2.89	1.04	1.00	5.00	0.857	6
PEUBI-Occult/Pseudoscience	2.60	0.88	1.00	4.91	0.838	11

Note. RWA-15 was scored on a 9-point scale (1–9); the total is the mean of the 12 items that form the three subscales. VSA-6 was scored on a centred 9-point scale (−4 to +4); subscale scores are means of the two constituent items. All Study 2 values reflect the corrected scoring described in Section 2.3.2. Reliability coefficients are Cronbach’s α; two-item subscales are reported with inter-item correlations in the text rather than α. k = number of items entering the score.

**Table 2:** Demographic baseline, aggregate-score, and alternative subdimension regression models predicting false-news accuracy (ACC_F, z-standardised) in Study 1.

Predictor	Coefficient	*SE*	*t*	*p*	95% CI
Demographic baseline—R^2^ = 0.042, F(4, 415) = 4.56, *p* = 0.001					
Aggregate-score model: + RWA Total—R^2^ = 0.228, ΔR^2^ = 0.186, ΔF(1, 414) = 99.84, *p* < 0.001					
Alternative subdimension model: RWA dimensions replacing RWA Total—R^2^ = 0.261, ΔR^2^ = 0.219, ΔF(3, 412) = 40.61, *p* < 0.001, f^2^ = 0.296					
Gender (woman = 1)	−0.016	0.091	−0.18	0.861	[−0.195, 0.163]
Age (z)	0.144	0.047	3.06	0.002	[0.052, 0.237]
Education (z)	0.034	0.047	0.74	0.462	[−0.057, 0.126]
Income (z)	0.067	0.050	1.35	0.178	[−0.031, 0.165]
RWA Aggression (z)	−0.075	0.049	−1.52	0.130	[−0.172, 0.022]
RWA Conventionalism (z)	−0.071	0.045	−1.57	0.117	[−0.160, 0.018]
RWA Submission (z)	−0.411	0.053	−7.78	<0.001	[−0.514, −0.307]

Note. For continuous z-standardised predictors, the reported coefficient is the standardized β. For gender, the coefficient is the adjusted woman-versus-man difference in outcome SD units. N = 420 complete cases. VIF range: 1.05–1.55. The aggregate-score and subdimension models are alternative expansions of the same demographic baseline; ΔR^2^ and F-change for the subdimension model are relative to that baseline.

**Table 3:** Demographic baseline, aggregate-score, and alternative component regression models predicting conspiracy-claim endorsement in Study 2.

Predictor	Coefficient	*SE*	*t*	*p*	95% CI
Demographic baseline—R^2^ = 0.116, F(4, 164) = 5.37, *p* < 0.001					
Aggregate-score model: + VSA Total—R^2^ = 0.347, ΔR^2^ = 0.232, ΔF(1, 163) = 57.82, *p* < 0.001					
Alternative component model: VSA components replacing VSA Total—R^2^ = 0.417, ΔR^2^ = 0.301, ΔF(3, 161) = 27.74, *p* < 0.001, f^2^ = 0.517					
Gender (woman = 1)	0.176	0.132	1.34	0.184	[−0.084, 0.437]
Age (z)	0.023	0.075	0.31	0.753	[−0.124, 0.171]
Education (z)	−0.185	0.070	−2.65	0.009	[−0.323, −0.047]
Income (z)	−0.001	0.083	−0.01	0.992	[−0.164, 0.163]
VSA Conservatism (Submission, z)	0.021	0.065	0.32	0.749	[−0.107, 0.149]
VSA Traditionalism (z)	0.529	0.066	7.99	<0.001	[0.399, 0.660]
VSA Authoritarianism (Aggression, z)	0.178	0.064	2.77	0.006	[0.051, 0.305]

Note. For continuous z-standardised predictors, the reported coefficient is the standardized β. For gender, the coefficient is the adjusted woman-versus-man difference in outcome SD units. N = 169 complete cases (one case excluded because of missing education). VIF range: 1.14–1.89. The VSA component model is exploratory because the VSA-6 was developed primarily as an overall RWA measure (Bizumic & Duckitt, 2018).

**Table 4:** Cross-study comparison of authoritarianism component effects (non-parametric bootstrap, 10,000 resamples).

Subdimension	β Study 1 (Flipped)	β Study 2	Δβ	95% CI
Aggression	0.075	0.178	+0.103	[−0.039, 0.241]
Conventionalism/Traditionalism	0.071	0.529	+0.458	[0.281, 0.621]
Submission/Conservatism	0.411	0.021	−0.390	[−0.545, −0.232]

Note. Coefficients are taken from the alternative subdimension/component models in Table 2 and Table 3. Study 1 coefficients are sign-reversed so that higher values denote greater susceptibility in both studies. Confidence intervals and *p* values derive from 10,000 independent bootstrap resamples.

## Data Availability

The data presented in this study are available on reasonable request from the corresponding author, subject to privacy and participant-confidentiality restrictions.
